# A decade of experience in the development and implementation of tissue banking informatics tools for intra and inter-institutional translational research

**DOI:** 10.4103/2153-3539.68314

**Published:** 2010-08-10

**Authors:** Waqas Amin, Harpreet Singh, Andre K. Pople, Sharon Winters, Rajiv Dhir, Anil V. Parwani, Michael J. Becich

**Affiliations:** 1 Department of Biomedical Informatics, University of Pittsburgh, Pittsburgh, USA; 2 Department of Pathology, University of Pittsburgh Medical Center, Pittsburgh, USA; 3 Registry information Service, UPMC Cancer Centers, Pittsburgh, PA. USA

**Keywords:** Tissue banking informatics, information models for translational research

## Abstract

**Context:**

Tissue banking informatics deals with standardized annotation, collection and storage of biospecimens that can further be shared by researchers. Over the last decade, the Department of Biomedical Informatics (DBMI) at the University of Pittsburgh has developed various tissue banking informatics tools to expedite translational medicine research. In this review, we describe the technical approach and capabilities of these models.

**Design:**

Clinical annotation of biospecimens requires data retrieval from various clinical information systems and the de-identification of the data by an honest broker. Based upon these requirements, DBMI, with its collaborators, has developed both Oracle-based organ-specific data marts and a more generic, model-driven architecture for biorepositories. The organ-specific models are developed utilizing Oracle 9.2.0.1 server tools and software applications and the model-driven architecture is implemented in a J2EE framework.

**Result:**

The organ-specific biorepositories implemented by DBMI include the Cooperative Prostate Cancer Tissue Resource (http://www.cpctr.info/), Pennsylvania Cancer Alliance Bioinformatics Consortium (http://pcabc.upmc.edu/main.cfm), EDRN Colorectal and Pancreatic Neoplasm Database (http://edrn.nci.nih.gov/) and Specialized Programs of Research Excellence (SPORE) Head and Neck Neoplasm Database (http://spores.nci.nih.gov/current/hn/index.htm). The model-based architecture is represented by the National Mesothelioma Virtual Bank (http://mesotissue.org/). These biorepositories provide thousands of well annotated biospecimens for the researchers that are searchable through query interfaces available via the Internet.

**Conclusion:**

These systems, developed and supported by our institute, serve to form a common platform for cancer research to accelerate progress in clinical and translational research. In addition, they provide a tangible infrastructure and resource for exposing research resources and biospecimen services in collaboration with the clinical anatomic pathology laboratory information system (APLIS) and the cancer registry information systems.

## INTRODUCTION

Tissue banking informatics is a new and relatively underdeveloped area of biomedical informatics that deals with the management of clinicopathologic annotation, inventory management and distribution of biospecimens that are collected and stored for translational research use by the scientific community. Annotation is a process of associating tissue samples with important demographic, epidemiology, pathology, tumor progression, vital status, therapy and outcome related data. This allows tissue samples to be matched with the research queries, thereby facilitating better understanding of the experimental design and result.

When individual research facilities collect data for their ongoing research, they follow their own protocols; therefore, the collected data are not uniform or shareable. It is critical to standardize the approach to annotation to ensure uniformity, consistency, and quality of collected data. This facilitates information sharing across multiple institutions. The development of an information model supported by common data elements (CDEs) and intelligent use of information technology to facilitate translational research are necessary for this purpose.[1]

One of the key foundational steps in the establishment of a tissue banking informatics program is establishing a standardized approach to the clinical annotation process. In previous work done at our institute, we have documented biorepository user data requirements and have identified seven areas of clinical annotations of key interest. These include patient demographics, histopathology, clinical staging data, laboratory data, tumor progression information, therapy and outcome data.[2] The source of data for clinical annotations is primarily obtained from several clinical systems including anatomic and clinical pathology laboratory information systems, electronic medical record systems and in the case of cancer research biorepositories, the cancer registry (CR) information systems. The process of clinical annotation is very dynamic and requires years of follow-up annotation to accurately reflect disease progression and patient outcome data.

## MATERIALS AND METHODS

### Architecture of Tissue Banking Information Systems

#### Clinical annotation

The key clinical systems mentioned above facilitate clinical annotation of biospecimens by providing detailed data that are collected by using CDEs along with patient and biospecimen identifiers. Patient enrollment data, biospecimen inventory, tissue quality assurance data and their availability for research studies are stored and managed in a database and should be presented as de-identified data to facilitate matching researchers’ requests for biospecimen availability.[3]

#### Anatomic pathology lab information system

The Anatomic pathology laboratory information system (APLIS) at University of Pittsburgh Medical Center (UPMC) Department of Pathology at University of Pittsburgh includes the CoPath Plus (Cerner, Waltham, MA, USA) for which we have co-developed with the vendor synoptic templates for reporting surgical pathology resection specimens. Synoptic reporting provides a structured method for entering the diagnostic as well as prognostic information for a particular pathology specimen or sample.[4,5] Using synoptic reports, consistent data elements with minimized typographical and transcription errors can be generated as discreet fields and placed in the laboratory information system (LIS) relational database, enabling quicker access to desired information and improved communication for appropriate cancer management. In addition to the diagnosis and management of the patient, the templates will also eventually serve as a medium for capturing and storing data in tissue banking information systems.[6–8]

#### Cancer registry information system via registry information services

The clinical and research registry tools are managed by the Research Registry Information Services (RIS), a division of the UPMC Cancer Centers, and constitute a valuable component of the University of Pittsburgh’s Cancer Institute’s (UPCI) Cancer Center Support Grant (CCSG). Standardized data are captured for all reportable diagnoses according to theNorth American Association of Central Cancer Registry (NAACCR) data standard.[9] Most facilities within the registry also hold to the voluntary standards set by the American College of Surgeons Commission on Cancer for approved cancer programs. Primary sources for data retrieval are both paper and electronic medical records and data are obtained by certified cancer registrars who also act as certified honest brokers.[10] Registry data include demographics, personal and medical history, diagnostic findings, primary cancer identification, staging, grading, treatment and outcomes. The RIS also manage a collaborative honest broker service.[10] These brokers are responsible to ensure Health Insurance Portability and Accountability Act (HIPAA) and Office of Human Research Protection (OHRP) of the Department of Health and Human Services (HHS) compliance for the release of information which involves data storing/storage in applications developed that are managed and utilized by researchers. Data from the CR system can be interfaced with the tissue banking information systems (TBIS), enabling the search of the registry data exclusively or in combination with research-specific data collected separately from Institutional Review Board (IRB)-approved research activities.

### Development of Common Data Elements

CDEs are clinical annotations that are defined in detail, utilizing metadata. These CDEs may be collected uniformly across multiple institutes, allowing sharing of data in a standardized format. Prior to developing a biorepository with standardized biospecimen annotations, there is significant amount of time, work, and commitment required from a multidisciplinary team including registrars, pathologists, oncologists, informaticians, technical and domains experts and the tissue bank professionals. At our institute, development of CDEs is managed and supervised by our pathologists in collaboration with our informatics team. This multidisciplinary team forms a CDE subcommittee with the responsibility to develop consensus CDEs (demographics, epidemiology, pathology – specimen as well as block level annotation, clinical phenotype, follow-up and outcome data) applicable to a variety of organ-specific tissue banking projects. In the process of developing CDEs, the subcommittee considers lessons learned from previous development efforts.[11–13] The major standards used to formulate the CDEs include the NAACCR data standards for CRs, the College of American Pathologists (CAP) cancer protocols and checklists, the Association of Directors of Anatomic and Surgical Pathology (ADASP) cancer reporting guidelines and the American Joint Committee on Cancer (AJCC) cancer staging manual.[9,14–16] Putting into practice these data standards, protocols, checklists and guidelines in the development of CDEs provides a powerful information model that facilitates both syntactic and semantic interoperability across multiple institutes.

### Honest Broker System and Human Subject Protection – Enabling the De-identification of Patient Health information

The clinically annotated biospecimens stored at various tissue banks employ decentralized sample and data collection and storage. Every case is assigned a unique identifier that is not linkable to clinical information systems or other protected health information. All specimens are collected on the basis of approved protocols from IRB and strict measures are taken to ensure that proper confidentiality and privacy of human subjects is protected in accordance with each of the participating institution’s IRB. The patient protected health information identifiers are maintained at the local institution and no links connecting to patient records are provided to the researchers. In addition, queries against our TBISs on publicly available portal (query) websites generate HIPAA compliant de-identified data sets (i.e. “Safe Harbor”) for the research community.[9,17]

University of Pittsburgh IRB and Office of Research Compliance facilitated the process for developing honest broker services and Department of Biomedical Informatics (DBMI) took the initiative in developing the first, cross-divisional, collaborative broker service,[10] in conjunction with our Health Sciences Tissue Bank and UPMC Network CR, to ensure compliance of a variety of tissue banks with specific regulatory agency guidelines for the release of Protected Health Information (PHI), including those of the OHRP of the Department of HHS, the HIPAA and the UPMC University of Pittsburgh IRB.[17]

### Implementation of Tissue Banking Information Systems

#### Data import and display in the tissue banking information system database

After collecting the tissue, pathologists review the surgical pathology report and select the key slides which represent the banked biospecimen, according to details provided by particular study protocols. Specimen (block) specific data elements are collected on the selected biospecimens and annotated in the TBIS. Tissue bank annotators (data entry clerks) and cancer registrars review and collect pathologic and clinical data on the selected cases. In most instances, this data entry is through web interfaces of the TBIS. Certain classes of data can be retrieved via interfaces from data sources like APLIS, and RIS into XML export files and then imported into the database through import scripts. The stored data are displayed to the end user through web-based query tools. The web pages are developed on static HTML images, style sheets and dynamically generated web pages through a variety of processes (see below) depending on the particular implementation of the TBIS.

### Tissue Banking Information Models and Architecture

There are two types of information models that are utilized in the development of tissue bank, which are described in this section.

#### Organ-specific databases

**Figure 1:**
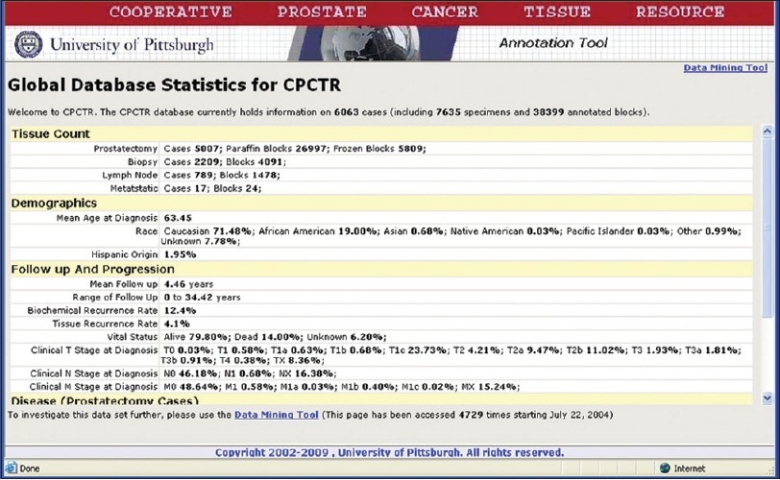
Image showing the CPCTR statistical database that is not password protected and allows users to query on stored de-identified annotated prostate cancer specimens. The statistical database provides overall statistics on user’s query on demographics, clinicopathology, follow-up and recurrence data sets

The organ-specific databases we have implemented include the Cooperative Prostate Cancer Tissue Resource (CPCTR), Pennsylvania Cancer Alliance for Bioinformatics Consortium (PCABC), Early Detection Research Network (EDRN) Colorectal and Pancreatic Neoplasm database and Specialized Program of Research Excellence (SPORE) Head and Neck Neoplasm Database [Figure 1]. The data warehouse is a three-tiered architecture and implemented on an Oracle Application Server v10.1.2.3 running on a Windows 2003 and Oracle RDBMS v.10.2.0.2 running on an AIX 5L virtual host definition which is supported by IBM ×3850 system hardware. The application utilizes the Oracle http server and mod_plsql extensions to generate dynamic web pages from the database to the users. The data annotation engine is a flexible dynamic web-based tool, while the data query engine facilitates investigators to search de-identified information within the warehouse through a “point and click” interface. The three layers consist of *Schema* layer which actually holds the physical data and data relations. All data are stored in numbers and keys. Middle layer, also referred to as *Meta* data layer, holds all data definitions and descriptions and relationships defined in terms of data elements and groups of data elements. Data descriptions such as data attributes, display attributes, valid values, DB Link, validation rules and documentation are supported in metadata. The metadata layer defines the application control layer. All user defined queries and results are controlled by internal/back end procedures/functions written using Oracle PL/SQL server pages and java scripts. The procedures accommodate changes in the metadata and immediately reflect the changes in the application layer. An application builder layer (form builder) is designed for data managers to define CDEs and their work flow and display criteria before the database administrator actually links the metadata layer to physical layer. The user management controls let the data manager to actually manage the various components within the database and control the application access for application users depending upon individual user privileges.[18]

#### Model driven approach

**Figure 2:**
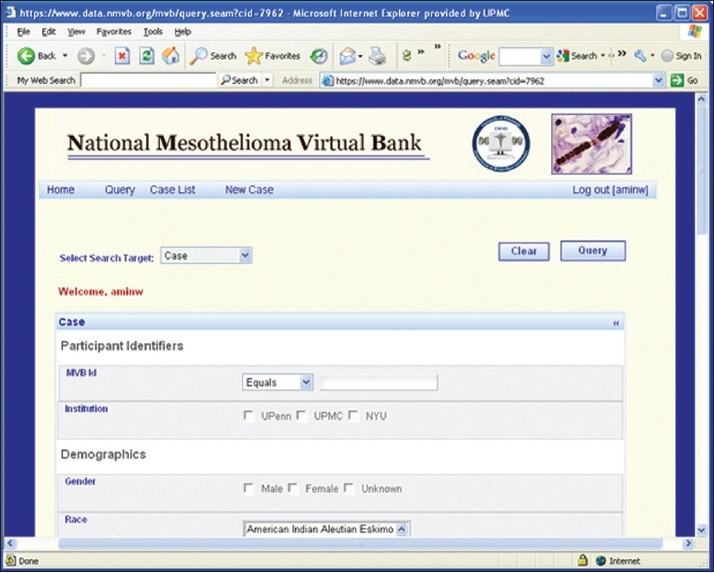
Image showing the NMVB password protected allows user to query interface. The database allows users to query on de-identified annotated mesothelioma specimens and in addition permits to view clinical data on each of the cases

The National Mesothelioma Virtual Bank (NMVB) is developed using a model-driven approach. The system is adapted from the caTISSUE Clinical Annotation Engine (CAE) system[19] that was developed by the University of Pittsburgh as a part of National Cancer Institute’s (NCI) caBIG program.[20] In a model-driven architecture, the developer, in consultation with domain experts, develops a Unified Model Language (UML) class diagram that represents the entities of the problem domain, their attributes and their relationships to each other. For the NMVB system, the Enterprise Architect (EA) tool (developed by Sparx System, Victoria, Australia)[21] was used as the UML modeling environment.[22] Once completed, the UML model is processed by the system to generate Java domain classes, a database schema and supporting metadata. These generated system components are incorporated into the model-driven framework to produce an operational data management system for managing the entities that are represented in the model [Figure 2].

The different components of the NMVB system are as follows: *Web Tier* provides static HTML, images, style sheets and metadata-driven Java Server Face (JSF) components that can construct web pages based upon the generated metadata. *Business Tier* consists of a set of functional components, an object/relational mapping mechanism, a metadata interrogation mechanism, an Application Programming Interface and a set of shared services. *Data Tier* consists of domain database that houses clinically annotated data, indexes to support the query mechanism and security data.

## RESULTS

**Figure 3:**
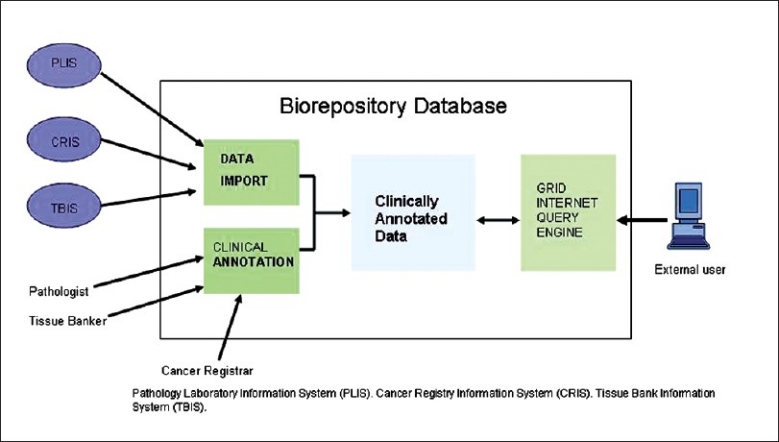
Image showing the architecture of biospecimen database with data import, integration and query capability in a web-based environment

We have developed multiple virtual biorepositories which were devised to standardize biospecimen-associated information so that individual collections of biospecimens can be shared by various research groups [Table 1]. The strategic vision is to ultimately enable automated systems interfaced to clinical cancer informatics environments to facilitate comprehensive phenotyping of cancer patients and their biospecimens. The components are devised in such a way that data can be integrated with the APLIS and RIS [Figure 3].

**Table 1:** Presents total number of cases enrolled and biospecimens’ availability in a variety of organ-specific and federated biorepositories

Virtual biorepository		Total number of cases	Total number of biospecimens		
			Paraffin blocks	Frozen blocks	Blood/serum/plasma
NMVB		844	306	255	670
CPCTR		7000	34641	17508	17508
PCABC	Breast	3645	1760	847	823
	Melanoma	1762	1885	168	112
	Prostate	7327	5457	1642	415
EDRN Colorectal and Pancreatic Neoplasm Virtual Biorepository		2227	175	942	1254
SPORE’s Head and Neck Neoplasm Virtual Biorepository		6553	2237	0	1038

MVB, Mesothelioma Virtual Bank; CPCTR, Cooperative Prostate Cancer Tissue Resource; PCABC, Pennsylvania Cancer Alliance for Biomedical Consortium; EDRN, Early Detection Research Network; SPORE, Specialized Program of Research Excellence

The multilayer data model/architecture for organ-specific biospecimen repositories facilitates collection of biospecimens and then incorporates these biospecimens with phenotyping data sets (clinical, epidemiologic, pathologic, treatment and follow-up, and genotypic molecular lab data) into a single web-based interface [Table 2]. The architecture of the organ-specific database is based on three components: a) Development of CDEs, which provides semantic and syntactic interoperability of the data sets by describing them in the form of metadata or data descriptor. b) The robust data entry tool is a portable and flexible Oracle-based data entry application is a web-based tool that is easy to use. c) The comprehensive data query tools with built-in de-identification logic facilitates investigators to search de-identified information within the warehouse through a “point and click" interface based on individual study based privileges. At the same time honest brokers can work on the identified data set without compromising the data privacy. This enables multiple researchers to independently do queries on individual data set.

**Table 2:** Comparison among different web-based tissue banking utilities for the research community developed at University of Pittsburgh

Human tissue repositories	Funding organizations	Information model	Data accessibility	User Web Interfaces		
				Public Statistical Query Interface	User Clinical database interface	Data entry interface plus electronic data import /export
NMVB (http://mesotissue.org/)	CDC/NIOSH	UML-model driven	U. Pitt, collaborators and IRB/SRCB approved Investigator	Yes	Yes	Both
PCABC (http://pcabc.upmc.edu/main.cfm)	Pennsylvania Department of Health	OSD	U. Pitt, collaborators and IRB/SRCB approved Investigator	Yes	Yes	Both
EDRN Colorectal and Pancreatic Virtual Biorepository	NCI	OSD	U. Pitt EDRN researchers only	No	Yes	Both
SPORE Head and Neck Neoplasm Virtual Biorepository	NCI	OSD	U. Pitt SPORE researchers only	No	Yes	Both
CPCTR (http://cpctr.info/)	NCI	OSD	U. Pitt, collaborators and IRB/SRCB approved Investigator	Yes	Yes	Electronic data import/export only

NMVB, National Mesothelioma Virtual Bank; PCABC, Pennsylvania Cancer Alliance Bioinformatics Consortium; CPCTR, Cooperative Prostate Cancer Tissue Resource; EDRN, Early Detection Research Network; SPORE, Specialized Program of Research Excellence; CDC, Center for Disease Control and Prevention; NIOSH, National Institute of Occupational Health and Safety; NCI, National Cancer Institute; OSD, Organ-specific database; U. Pitt, University of Pittsburgh; IRB, Institutional Review Board; SRCB, Scientific Review Committee Board

The model-based approach used for the NMVB system also incorporates CDEs (based on CAP protocol and NAACCR standards), a web-based data entry application, and data query tools. The metadata is captured in a UML model and associated XML-formatted files.[22,23]

## DISCUSSION

The increasing demand of intra-institutional and cross-institutional translational research and biospecimens has fueled the development of advance tissue banking informatics tools with a capability of providing the research community with superior quality, well annotated biospecimens. To fulfill the research community’s requirements, over the last decade, our institute has developed and successfully implemented various organ- and disease-specific tissue banking tools. These TBIS are constructed on an underlying architecture of CDEs for enriched characterization of tissue samples and clinical follow-up data which are supported by an integrated quality assurance process.

The development and implementation of these tools has enabled an enhanced utilization of the annotated biospecimens by the cancer research community. In addition, through the development of these systems, we have implemented mechanisms to make these valuable biospecimens more broadly available to the research community. These tools also support a workflow process for investigators to obtain tissue samples and phenotypic data. The request process utilizes an independent research evaluation panel (REP) which is assisted by biostatisticians and pathologists to efficiently fulfill biospecimen requests.

In order to provide data about patients who utilize multiple health care systems, it is necessary to incorporate the patient data from various sources. One of the fundamental functionalities of the tissue bank informatics tools is to accurately identify the patient (without duplication or misidentification) across multiple data sources. This is achieved by locating a single unique patient identifier that is common in all multiple data source systems. This unique identifier is verified at the primary data sources as part of our quality assurance processes. After identifying the same patient across multiple data sources, the individual data elements are mapped to the CDEs and are then incorporated into the database by using integral mapping applications.

The data collection errors can occur even with careful attention to integrate multiple sources of data for the same patient or the absence of specific identifiers unique to the patients. A manual review is necessary to address these mismatch errors and to maintain data quality.

Secondly, in order to maintain validity of data for each patient, the collected data with temporal relationships still must be evaluated for context before they can be captured into a database. This approach was carried out by the resource to handle data context that is basically a manual process of data collection and entry. Furthermore, this method allows incorporation of additional data elements than may be needed for the resource, including data elements not relevant to the biorepository. These additional data elements provide integration with other data sources and serve to verify the data integrity. Despite the effectiveness of these measures, the process may be hampered by the presence of unstructured systems using free text *in lieu* of structured data fields, thereby complicating searching capabilities within these databases. In this scenario, manual annotation may be utilized to resolve this issue.

De-identification is the process which includes removal of PHI from patient clinical data annotations received from APLIS, CR, Electronic Medical Record (EMR) and chart reviews by data entry personnel and CR staff. This process is done by honest brokers who act as a bridge between clinical identifiable data sets, limited data sets and fully de-identified research data sets (HIPAA “Safe Harbor”) both via manual efforts as well as by utilizing a tool called De-ID (De-ID Data Corp, Richboro, PA, USA).[24] At our institute, our IRB has certified the use of our de-identification software and workflow through our Honest Broker System.[25] The honest broker collects clinical data and/or biological materials by the nature of their daily clinical responsibilities, identifies the patient based on any number of parameters, and separates cases relevant to a specific project by assigning each case a research project-associated de-identification “linkage codes” which may consist of numbers, letters, characters or any combination therein. The cross-reference of patient identifiers to de-identification code is only available to the “honest broker” or brokers associated with the project to permit information collation and/or subsequent inquiries. Only the de-identified data set is made available to the research community.

Although over the last decade, much work has been done in managing informatics-related challenges, such as correlating a single patient with multiple records from a variety of clinical systems, there remains a need for human interaction in this process that the “honest brokers” must fulfill. Honest brokers are the insurance mechanism for HIPAA compliant use of de-identified clinical data in the IRB-exempt research domain. Their role also ensures validation of automated informatics solutions integrating patient-related data from the various clinical systems. Finally, brokers must document the use of such services to complement auditing requirements of honest broker systems.

To facilitate the standardized clinical annotation process of biospecimens and to automate the process of annotation, the CDEs are developed for each tissue resource by a designated CDE subcommittee. Open discussions and input from all potential parties with a stake in the outcome are decisive to any development of tissue biorepositories. It is very important to include the tissue bankers, data managers, and cancer registrars in CDE development, who are the main data collectors for the tissue banking resource. Their input on the types of data and data descriptors available for collection proved to be vital in aggregating high-quality annotation data for the biospecimens.

The previously described process of developing the CDEs has been validated that this approach can successfully lead to the implementation of robust human tissue biorepositories-related CDEs that guide the collection of high-quality data for the research community while attempting to project at least 5 years into the future for additional data that may become clinically significant.

The aforementioned information model for organ-specific tissue banking efforts such as prostate cancer, head and neck neoplasms, colon and pancreatic malignancies provides highly effective infrastructure that allows for proficient control, standardized attainment of data and comprehensive annotation of cases. Utilization of an Oracle database model for query tool development has resulted in the production of tiered web-based query tools. This has facilitated the sharing of data between the collaboration as well as authorized investigators. *Per se* the resource offers an important knowledge base for the growth of integrated tissue banking programs. These specific query tools boast of swift performance, vigorous security utilities, and expansion potential for accommodating new data elements or incorporating presently available system functions.

The information model for model driven database is adopted from the caTissue clinical annotation engine (CAE) which was an integral component of the CaTissue suite developed as part of the CaBIG project. The tool aids in developing and conveying the semantic interoperability of the data system, by describing the CDEs in the form of metadata or data descriptors (about the content, quality, condition, and other characteristics of the data) and by using controlled vocabulary and ontology, in order to make the data understandable and sharable for end-users and flexible for the system.

Each CDE is associated with an object or concept, attribute, and valid value(s). Although the concept of formalized metadata is fairly straightforward, it has rarely been incorporated by clinical and research groups in building databases. The advantages of these CDE-based systems over other approaches include their ability to exchange information in a common format and the capacity to understand and use the information once it is received by other systems and provision for automated transfer of data from the source databases [Tissue Bank Inventory System (TBINV), RIS, coPath Plus].

## CONCLUSION

There are several challenges in setting up a tissue banking informatics infrastructure to support cancer research. The collection of tissue, development and implementation of CDE standards, clinical annotation, de-identifying the data sets, unique identification of patients across different data sources and development of data query tools for external use requires significant challenges. The successful development of these tools at our institute over the last decade has relied upon the significant interactions with the APLIS, CPLIS and CR to allow for the enriched clinical annotation of these biospecimens. The two models currently deployed at our institute have greatly facilitated the sharing of biospecimens and clinical phenotyping data to significantly enable translational research. Future efforts will focus on more powerful tools of interoperability to bring the data from multiple institutions in alignment with each other so that cross-institutional research efforts are more successful.
